# Deletion of *Fmr1* Alters Function and Synaptic Inputs in the Auditory Brainstem

**DOI:** 10.1371/journal.pone.0117266

**Published:** 2015-02-13

**Authors:** Sarah E. Rotschafer, Sonya Marshak, Karina S. Cramer

**Affiliations:** Department of Neurobiology and Behavior, University of California Irvine, Irvine, California, 92697, United States of America; University of Washington, Institute for Stem Cells and Regenerative Medicine, UNITED STATES

## Abstract

Fragile X Syndrome (FXS), a neurodevelopmental disorder, is the most prevalent single-gene cause of autism spectrum disorder. Autism has been associated with impaired auditory processing, abnormalities in the auditory brainstem response (ABR), and reduced cell number and size in the auditory brainstem nuclei. FXS is characterized by elevated cortical responses to sound stimuli, with some evidence for aberrant ABRs. Here, we assessed ABRs and auditory brainstem anatomy in *Fmr1* ^-/-^ mice, an animal model of FXS. We found that *Fmr1* ^-/-^ mice showed elevated response thresholds to both click and tone stimuli. Amplitudes of ABR responses were reduced in *Fmr1* ^-/-^ mice for early peaks of the ABR. The growth of the peak I response with sound intensity was less steep in mutants that in wild type mice. In contrast, amplitudes and response growth in peaks IV and V did not differ between these groups. We did not observe differences in peak latencies or in interpeak latencies. Cell size was reduced in *Fmr1* ^-/-^ mice in the ventral cochlear nucleus (VCN) and in the medial nucleus of the trapezoid body (MNTB). We quantified levels of inhibitory and excitatory synaptic inputs in these nuclei using markers for presynaptic proteins. We measured VGAT and VGLUT immunolabeling in VCN, MNTB, and the lateral superior olive (LSO). VGAT expression in MNTB was significantly greater in the *Fmr1* ^-/-^ mouse than in wild type mice. Together, these observations demonstrate that FXS affects peripheral and central aspects of hearing and alters the balance of excitation and inhibition in the auditory brainstem.

## INTRODUCTION

Fragile X syndrome (FXS) is the most common single-gene inherited form of autism. FXS results from an expansion of the CGG repeats in the promoter region of the *FMR1* gene, which reduces the amount of fragile X mental retardation protein (FMRP) produced. FMRP acts as a modulator of mRNA translation and has numerous target genes [1, 2]; its loss results in unregulated production of synaptic proteins [3]. Individuals with FXS display cognitive impairments, hyperactivity, seizures, aberrant dendritic spine morphology and several autism-related symptoms [4–8].

FXS subjects also display a general enhancement of response to sensory stimuli. When presented with an array of stimuli that spanned several sensory modalities, individuals with FXS show elevated electrodermal responses across all types of stimuli [9, 10]. Notably, there is a heightened response to auditory stimuli. When subjects with FXS were presented with sound stimuli as part of an oddball discrimination task, cortical responses were consistently elevated [11–15]. Whether heightened cortical response in FXS is a phenomenon unique to the cortex or results from dysfunction of other auditory brain structures is unknown. However, electrophysiological and anatomical evidence suggest impaired auditory brainstem function. Auditory brainstem responses (ABRs) performed on individuals with FXS have reported longer absolute peak latencies and inter-peak intervals [16–19]. Of particular interest, prolonged I-III interpeak interval (Ferri et al., 1986) and III-V interpeak interval (Arinami et al., 1988) have been reported in FXS. Peak III results from the activation of the superior olivary nuclei in response to sound; therefore altered peak III latency may suggest some dysfunction of the superior olivary complex in FXS patients. Other studies attribute ABR anomalies in FXS to impaired peripheral auditory processing [20], or sedatives which may have been used during the testing process [21].

In addition to these physiological observations, molecular and anatomical changes have been associated with the auditory brainstem in FXS. Aberrations in brainstem anatomy have been found in the post mortem tissue of subjects with FXS and autism [22, 23]. A survey of autistic men and boys revealed reduced cell number in medial superior olive (MSO) of several subjects. Autistic MSO cell bodies were smaller and more variable in their orientation than in controls. Of note, an individual included in this study who was diagnosed with FXS and autism also displayed the MSO anomalies found in his autistic counterparts [22]. Other work examining the auditory brainstem of autistic individuals found a 77% decrease in the number of MSO neurons, a 67% decrease in the number of lateral superior olive (LSO) neurons, and a 45% decrease in the number of medial nucleus of the trapezoid body (MNTB) neurons as well as altered composition of neuronal populations within nuclei [24]. Within the MNTB, the Kv3.1b potassium channel is typically expressed in a tonotopic gradient. In FXS, the Kv3.1b gradient is lost, leading to improper coding of sound stimuli and impaired sensory modulation in the MNTB [25]. Additionally, FMRP is present in 83% of MSO cells and has been found to concentrate at dendritic branch points of MSO cells across species [26, 27]. Excessive dendritic branching and impaired dendritic pruning have been reported in animal models of FXS [28, 29].

In this study, we examined ABRs and quantified excitatory and inhibitory inputs to auditory brainstem nuclei in the *Fmr1* ^-/-^ (KO) mouse, an animal model of FXS. Similar to individuals with FXS, *Fmr1* ^-/-^ mice have aberrations in dendritic spine number and morphology, hyperactivity, audiogenic seizures, repetitive behavior, communication deficits, and difficulties with social interactions, making them a useful model for studying FXS-related dysfunction [30–33]. Our results suggest that loss of *Fmr1* causes reduction in the ABR response, with both central and peripheral components. *Fmr1* ^-/-^ mice displayed elevated ABR thresholds and showed reduced growth of responses with stimulus intensity compared to WT animals. We found that VCN and MNTB cell size was decreased in *Fmr1* ^-/-^ mice compared to wild type animals, and MNTB showed increased expression of vesicular GABA transporter protein (VGAT) relative to vesicular glutamate transporter protein (VGLUT) expression within the MNTB.

## MATERIALS AND METHODS

### Animals

Adult male *Fmr1* ^-/-^ mice and wild type littermates on the FVB strain were used for ABR measurements and anatomical studies. All procedures were approved by the University of California, Irvine Institutional Animal Care and Use Committee.

### ABR Recordings

ABRs were performed inside a 102cm x 98cm x 81cm sound-attenuating chamber (Industrial Acoustics Company, NY). Before recording ABR responses, the ABR system was calibrated under computer control from 5kHz to 80kHz in 5Hz steps using a 0.5-inch condenser microphone model no. 4134 (Bruel & Kjaer). Mice were anesthetized with a solution of ketamine (75–85mg/kg) and xylazine (0.1–0.5 mg/kg) and placed on a Kopf Model 900 Small Animal stereotaxic instrument (Tujunga, CA). Body temperature was monitored and maintained with a custom built heating pad. Sound was presented using a Tucker-Davis Technologies (TDT) MF1 Multi-Function Speaker (Alachua, FL) through an ear tube placed in animals’ left ear. Pin electrodes were placed subdermally to probe brainstem responses. The positive (active) electrode was placed at the vertex, the negative (reference) electrode was placed in the right cheek, and a ground electrode was placed at the base of the tail. The pin electrodes were connected to a TDT RA4PA 4-channel Medusa preamplifier, which connected to a TDT RA16 Medusa Base Station.

Sound stimuli were generated using Tucker-Davis Technologies SigGen software and presented under the control of the TDT BioSig platform. Presentation of sound stimuli was driven by a TDT RP2.1 enhanced real time processor and signal level was controlled using a TDT PA5 programmable attenuator. Sound was amplified by a TDT SA1 stereo power amp. All sounds were presented 500 times at a rate of 29 stimuli per second. Mice were first presented with 100 μs click stimuli between 80 and 10 dB SPL, decreasing in 5 dB SPL steps. Next, mice were presented with 8, 12, 16, 24, and 32 kHz tone stimuli. Each tone was 3ms long and sound intensity was reduced in 5 dB SPL intervals, beginning at 80 dB SPL and ending at 10 dB SPL.

### ABR Analysis

ABR traces were analyzed for threshold, peak latency, peak amplitude, and inter-peak interval in response to click and tone stimuli. Threshold was defined as the lowest intensity at which any peaks could be discerned. Peak latency was the time from the onset of a stimulus to the time at which each peak reached its apex. Peak amplitude was measured as the voltage at the apex of each peak relative to the noise floor. The inter-peak interval was the time between the apex of one peak and the apex of another peak.

To test for genotype differences in peak latency and peak amplitude, latency and amplitude data were collected for peaks I, II, III, and IV. We analyzed input-output functions to compare WT and KO animals in their response amplitudes (μV) and latencies (msec) as sound intensity (dB SPL) increased. We performed two-way repeated measure ANOVA (JMP 9.0, SAS Institute, Cary, NC) on each peak to determine the effect of genotype and sound intensity and to identify interactions between these variables.

### Nissl Staining and Analysis

Deeply anesthetized animals were perfused with 0.9% saline followed by 4% paraformaldehyde in phosphate buffered saline (PBS) before brainstems were harvested. Dissected brainstem specimens were post-fixed with 4% paraformaldehyde for at least 2 hours, incubated overnight in a 30% sucrose solution in PBS, and sectioned in the coronal plane at 16 μm on a cryostat. Sections were collected on chrome-alum coated glass slides in a 1-in-10 series and dried on a 37^o^ C slide warmer. Mounted sections from one set of alternate sections were stained with 1% thionin and coverslipped. The ventral cochlear nucleus (VCN), MNTB, and LSO were digitally imaged on a Zeiss Axioskop2 microscope using Axiovision acquisition software.

To determine cell density in the VCN and LSO, a 9530 μm^2^ outline was placed over a portion of a VCN or LSO image in a minimum of three sections per brain. ImageJ cell counter was used to count all cells within the outline. The number of cells was divided by the area of the outline to find cell density. Cell density in MNTB was found by placing a 14405 μm^2^ outline over the medial MNTB and lateral MNTB. The number of cells in both the medial and lateral outlines were counted using ImageJ cell counter and divided by the area of the outline to find cell density. Cell densities were found in both the left and right VCN and MNTB. Left and right values were averaged to produce final VCN, medial MNTB, and lateral MNTB cell densities.

To determine cell area, outlines of Nissl labeled cells in each nucleus were analyzed in ImageJ. At least 10 cells were measured in each section, using a minimum of three sections distributed through the rostrocaudal extent of the nucleus. Cells were selected for measurement if the nucleus was discernable within the section. Cross sectional area was recorded and cell areas were averaged to obtain a single value for each nucleus for each brain.

### VGLUT and VGAT Immunostaining

Cryosectioned brainstems were used for immunofluorescence. Mounted sections were surrounded with a hydrophobic Pap pen barrier and rinsed. For antigen retrieval we placed slides in 0.1% sodium dodecyl sulfate in PBS for 5 minutes. Slides were then rinsed with PBS and incubated at room temperature in a humid chamber with blocking solution (4% bovine serum albumin, 0.4% Triton X in PBS) for 1 hour. Slides were then incubated with primary antibodies diluted in blocking solution. We used a rabbit polyclonal antibody that recognizes VGAT (Phosphosolutions, Aurora, CO) at 1:200 and a guinea pig polyclonal antibody that recognizes VGLUT2 (Millipore, Temecula, CA) at 1:2500. Slides were washed in PBS then incubated at room temperature for 2 hours in AlexaFluor (Invitrogen, Carlsbad, CA) secondary antibodies (goat anti-guinea pig Alexa 594 and goat anti-rabbit Alexa 488) diluted 1:500 in blocking solution. Slides were rinsed in PBS, and coverslipped using ProLong Gold antifade reagent with DAPI (LifeTechnologies, Eugene, OR) as a mounting medium.

### Imaging and Analysis

Slides were viewed on a Zeiss AxioSkop-2 epiflourescence microscope. Images of regions containing the VCN, LSO and MNTB were processed using ImageJ software. For this analysis we included at least three sections spanning the rostrocaudal extent of each nucleus. We outlined the nuclei and found the total area immunopositive for VGLUT and VGAT. Using ImageJ, images were separated by red (VGLUT), green (VGAT), and blue (DAPI) color-channels. The thresholds on the red and green channels were adjusted to highlight the stained portions of the image on each channel. The ImageJ Analyze Particles function was used to summate all of the VGLUT- or VGAT-stained objects of an image. We defined an index (I_SP_) to characterize relative levels of each synaptic protein for each nucleus: I_SP_ = (VGLUT-VGAT)/(VGLUT+VGAT)

Values of I_SP_ range between -1 and 1. Positive values of I_SP_ indicate more excitatory puncta than inhibitory puncta, and negative values would indicate more inhibition. Absolute values of I_SP_ reflect differences in the relative levels of these two proteins. To determine statistical significance for anatomical data, we performed one-way ANOVA with post-hoc two-tailed t-tests using Bonferroni correction to adjust for multiple comparisons.

## RESULTS

### ABR measurements

**Fig 1:**
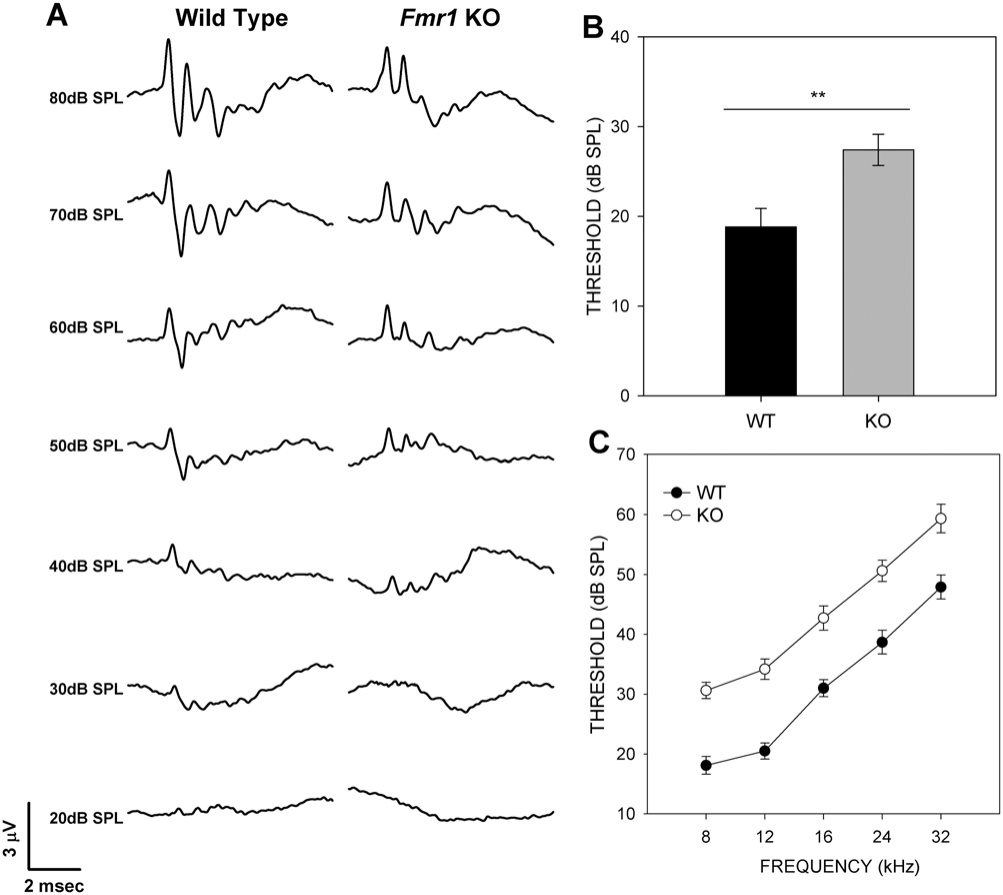
*Fmr1* ^-/-^ mice have higher hearing thresholds in response to click and pure tone stimuli. (A) Representative ABR traces in response to click stimuli from wild type mice (left) and *Fmr1* ^-/-^ mice (right). (B) ABR thresholds are significantly higher in *Fmr1* ^-/-^ mice (gray) than in wild type mice (black) in response to clicks. (C) *Fmr1* ^-/-^ mouse ABR thresholds were significantly higher in response to pure tone stimuli then those of wild type mice.

A total of 25 *Fmr1* ^-/-^ mice and 20 wild type mice were used for ABR measurements. Click or pure tone stimuli were presented at intensities beginning at 80 dB SPL decreasing in 5 dB SPL steps to 10 dB SPL (Fig. 1A). Traces generated in response to both types of stimuli were analyzed for threshold, peak amplitude, absolute peak latency, and inter-peak latency. Peak V could be identified after click stimulation but not in most traces generated using tone stimuli.

### ABR thresholds are elevated in KO mice

The mean threshold for the ABR in KO mice was 27.5 ± 1.88 dB SPL (± SEM, n = 24), which was significantly higher than those of WT mice when tested with clicks (18.5 ± 2.06 dB SPL (n = 20; two-tailed t-test, p < 0.0024; Fig. 1B). KO mice also had elevated thresholds compared to controls at each of the five pure tone stimuli tested (Fig. 1C; Table 1), with significance ascertained after Bonferroni correction.

**Table 1:** Analysis of ABR mean thresholds.

**Sound stimulus**	**Mean threshold KO, dB SPL (n)**	**Mean threshold WT, dB SPL (n)**	***p***
*Click*	27.5 ± 1.88 (24)	18.5 ± 2.06 (20)	0.0024*
*8 kHz*	30.6 ± 1.37 (24)	18.1 ± 1.47 (21)	0.0001*
*12 kHz*	34.2 ± 1.50 (24)	20.5 ± 1.64 (20)	0.0001*
*16 kHz*	42.7 ± 1.73 (24)	31.0 ± 1.90 (20)	0.0001*
*24 kHz*	50.6 ± 1.78 (24)	38.7 ± 2.00 (19)	0.0001*
*32 kHz*	59.3 ± 2.14 (23)	47.9 ± 2.36 (19)	0.0001*

### ABR amplitude

**Fig 2:**
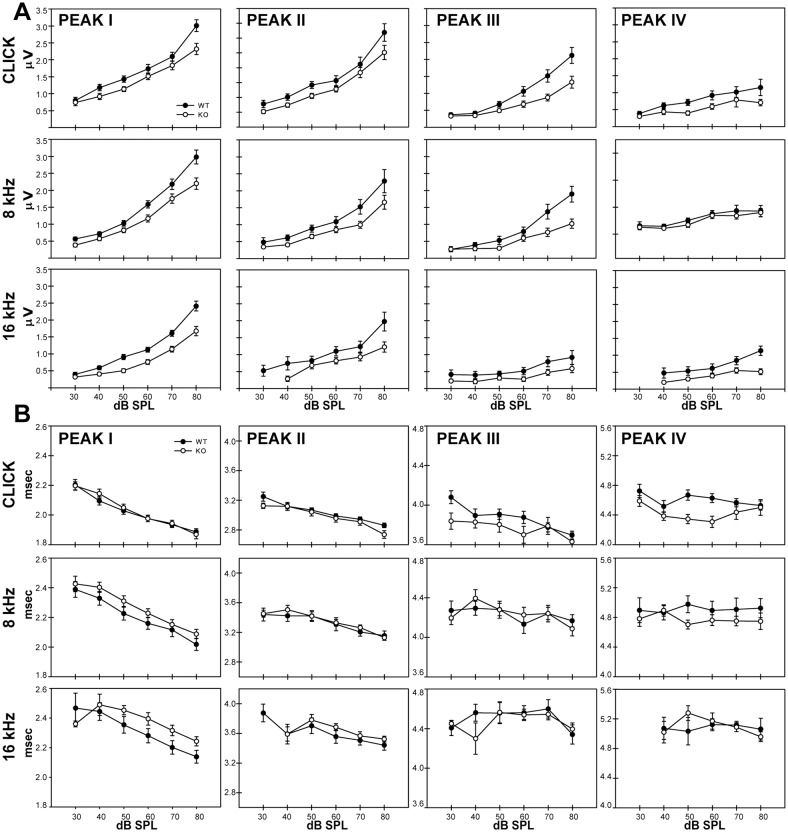
Input-output functions in response to click, 8kHz, and 16kHz stimuli for peak amplitude (A) and peak latency (B). Peak I is represented by the furthest left column, peak II by the middle left column, peak III by the middle right column, and peak IV by the furthest right column. Significant differences in peak amplitude (A) were found between wild type (black circles) and *Fmr1* ^-/-^ (open circles) at peaks I and III. Few significant differences were found in peak latency (B).

To assess how peak amplitude may change with stimulus intensity, we generated input-output functions for each peak, where the mean peak amplitude was plotted against stimulus intensity (Fig. 2). Peak latency and amplitude were analyzed for each peak for intensities at 40, 50, 60, 70, and 80 dB SPL following click stimuli. For peak I, two-way repeated measure ANOVA revealed a significant effect of genotype on peak amplitude (F_1,40_ = 5.94, *p* = 0.0193) and also showed a significant interaction between genotype and sound intensity (F_4,37_ = 3.98, *p* = 0.0087). The peak I responses in *Fmr1* ^-/-^ mice were smaller than those of wild type mice and increased more slowly with sound intensity. We next tested whether this effect was evident using pure tone stimuli. Genotype significantly reduced peak I responses to stimuli at 8 kHz (F_1,39_ = 6.76, *p* < 0.0131), 12 kHz (F_1,34_ = 11.363, *p* < 0.0019), and 16kHz (F_1,25_ = 10.88, *p* < 0.0029). The interaction between genotype and intensity was also seen for 12 kHz stimuli (F_4,31_ = 2.79, *p* < 0.0433); *p* values for all comparisons are shown in S1 Table. The effects for tone stimuli further support effect of *Fmr1* deletion on decreased peak I amplitude and gain.

Analysis of peak II following click stimuli did not show an effect of genotype on peak response (F_1,40_ = 4.0428, *p* = 0.0511) and no interaction between genotype and sound intensity (F_4,37_ = 0.8590, *p* = 0.498). Consistent with this observation, effect of genotype did not reach significance for any of the tone frequencies presented (S1 Table). Peak III amplitude was significantly reduced in KO mice when click tones were presented (F_1,33_ = 9.6590, *p* = 0.0039) and when 8 kHz tone stimuli were used (F_1,17_ = 8.0925, *p* = 0.0112), but not at other frequencies. The interaction between genotype and sound intensity did not reach significance for peak III amplitude. We did not see effects of genotype on peak amplitude for click stimuli for peak IV or peak V, and no effect of interaction between genotype and sound intensity was seen (S1 Table).

### ABR latency

We examined how peak latency varied with stimulus intensity in wild type and *Fmr1* ^-/-^ mice. Two-way repeated measure ANOVAs revealed no main effect of genotype on peak latency for any peak using click stimuli. Responses to tone stimuli were largely similar. Significant effects were seen only in two cases: 16 kHz stimulation at peak II and 12 kHz stimulation at peak III (S1 Table), and interpeak measurements of latency did not show main effects of genotype for any stimulus.

### Histological analysis of auditory brainstem nuclei

**Fig 3:**
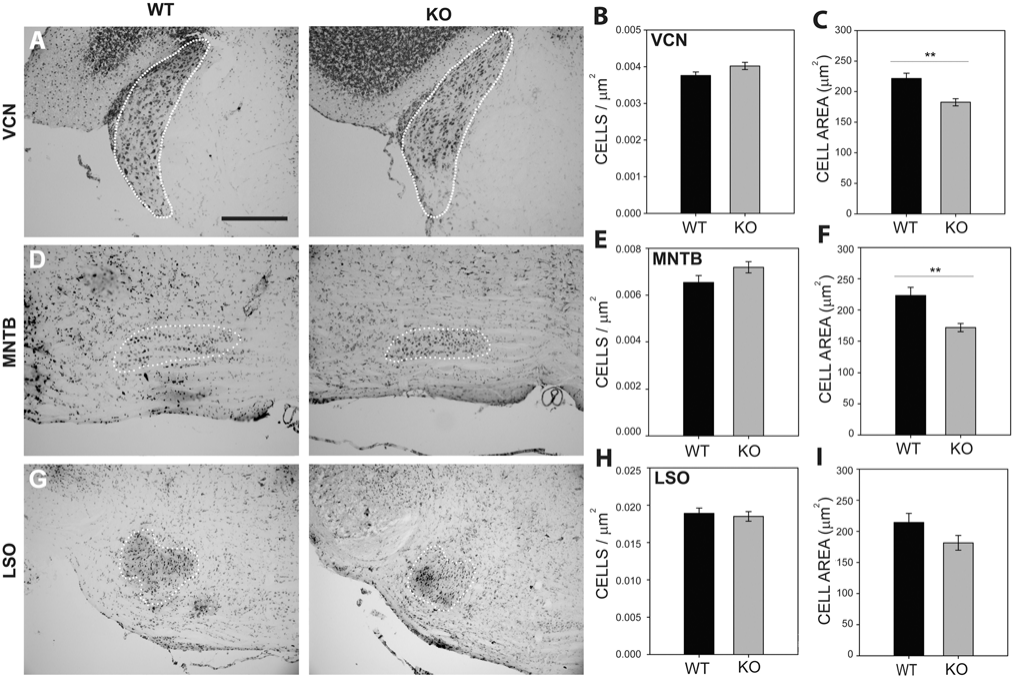
Nissl staining revealed a significant decrease in VCN and MNTB cell area in *Fmr1* ^-/-^ mice. Nissl stains were performed on mouse brainstem tissue and the borders of VCN (A), MNTB (D), and LSO (G) were identified in wild type and *Fmr1* ^-/-^ mice. Cells were counted within these regions and used to calculate cell density for each nucleus. No significant differences in cell density were found in VCN (B), MNTB (E), or LSO (H). Cell area was significantly reduced in *Fmr1* ^-/-^ mice in VCN (C) and MNTB (F), but not in LSO (I). Scale bar in A, 200 μm; applies to A, D, and G.

To probe anatomical anomalies potentially related to central components of the ABR changes seen in *Fmr1* ^-/-^ mice, we performed histological analysis on Nissl stained brainstem sections. The VCN, MNTB, and LSO of 7 wild type and 7 *Fmr1* ^-/-^ mice were analyzed for cell density and cell area (Fig. 3I). We found no significant differences in cell density within the VCN (WT = 0.004 ± 0.00016 cells/μm^2^, KO = 0.004 ± 0.00029, cells/μm^2^; *p* = 0.704; Fig. 3B), MNTB (WT = 0.0063 ± 0.00033 cells/μm^2^, KO = 0.0069 ± 0.001 cells/μm^2^; *p* = 0.2494; Fig. 3E), or LSO (WT = 0.019 ± 0.001 cells/μm^2^, KO = 0.019 ± 0.001cells/μm^2^; *p* = 0.8872; Fig. 3H).

Cell area measurements showed significant reduction in cell size in VCN and MNTB, but not in LSO. In VCN (Fig. 3C) the mean cell area for wild type mice was 222 ± 8.7 μm^2^ (n = 8) while the area for *Fmr1* ^-/-^ mice was 183 ± 5.8 μm^2^ (n = 9; two-tailed t-test with Bonferroni correction, *p* = 0.0017). In MNTB (Fig. 3F) the mean cell area for wild type mice (223 ± 13 μm^2^, n = 9) was greater than that for *Fmr1* ^-/-^ mice (172 ± 6.6 μm^2^, n = 7; *p* = 0.002). In LSO (Fig. 3I), wild type cell area (214 ± 15 μm^2^, n = 8) was similar to that seen in *Fmr1* ^-/-^ mice (182 ± 12 μm^2^, n = 8; *p* = 0.102).

### Inhibitory and excitatory synaptic proteins in the auditory brainstem nuclei

**Fig 4:**
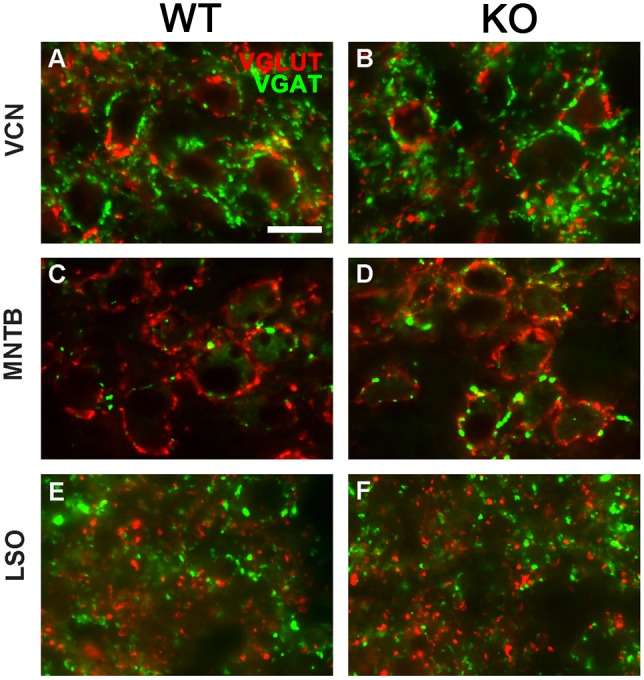
Wild type (left column) and *Fmr1* ^-/-^ (right column) brainstem tissue was stained for markers of excitatory and inhibitory inputs. VCN (A-B), MNTB (C-D), and LSO (E-F) were stained for VGLUT (red) and VGAT (green). Scale bar in A, 50 μm; applies to all panels.

To estimate relative levels of synaptic input in the auditory brainstem, we performed double immunofluorescence for VGLUT and VGAT. We quantified the area of VGLUT-positive and VGAT-positive inputs to cells in the VCN, MNTB, and LSO. Regions of VCN (Fig. 4A–B), MNTB (Fig. 4C–D), and LSO (Fig. 4E–F) were selected, and thresholds adjusted such that the total area of VGLUT or VGAT within a region was highlighted, and the label density, or fractional areal coverage, was computed by dividing the area with label by the area of the sampled region.

**Fig 5:**
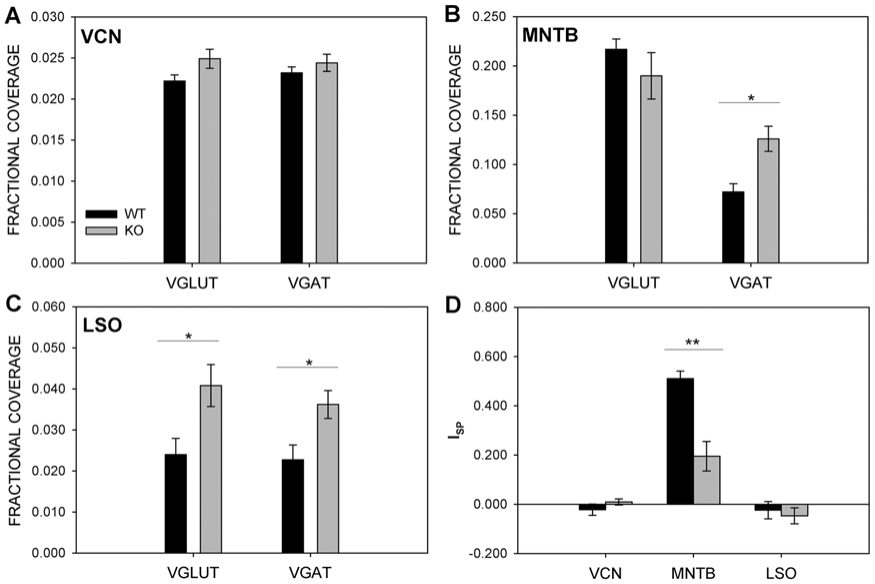
*Fmr1* ^-/-^ MNTB cells receive significantly more GABAergic input. The fractional coverage of VGLUT and VGAT was assessed in the VCN (A), MNTB (B), and LSO (C). No significant differences were found in VGLUT or VGAT fractional coverage in VCN (A). *Fmr1* ^-/-^ (gray) MNTB had significantly more VGAT coverage than wild type MNTB (black), though there was no difference in VGLUT coverage. VGLUT and VGAT fractional coverage was significantly greater in *Fmr1* ^-/-^ within the LSO. VGLUT and VGAT fractional coverage was compared by calculating a synaptic protein index value for each nucleus (D). No significant differences were found in VCN or LSO, but *Fmr1* ^-/-^ MNTB had lower index values than wild type MNTB. The smaller I_SP_ value likely reflects the heightened VGAT staining in *Fmr1* ^-/-^ MNTB.

In VCN the fractional coverage of VGLUT did not differ between wild type mice (0.22 ±.00074, n = 8) and *Fmr1* ^-/-^ mice (0.025 ± 0.00116, n = 10; *p* = 0.0913). Similarly, VGAT did not differ between wild type mice (0.023 ± 0.0007, n = 8) and *Fmr1* ^-/-^ mice (0.024 ± 0.001, n = 10; *p* = 0.3919) in VCN (Fig. 5A). In MNTB VGLUT density was similar in wild type mice (0.217 ± 0.01, n = 9) and *Fmr1* ^-/-^ mice (0.19 ± 0.019, n = 11; *p* = 0.3559). However, MNTB showed a significant increase of VGAT in *Fmr1* ^-/-^ mice (0.126 ± 0.013, n = 11) compared to wild type controls (0.072 ± 0.008, n = 9; *p* = 0.0034) (Fig. 5B). In LSO, the increase in VGLUT and VGAT density in *Fmr1* ^-/-^ mice was not significant after Bonferroni correction (VGLUT: WT, 0.023 ± 0.0036, n = 7; KO 0.036 ± 0.0034, n = 9; *p* = 0.0176; VGAT: WT, 0.024 ± 0.0040, n = 7; KO, 0.041 ± 0.005, n = 9; *p* = 0.0272; Fig. 5C).

Thresholded VGLUT and VGAT regions were then summated and an index I_SP_ comparing VGLUT to VGAT within each region tested was calculated (Fig. 5D). No significant differences were seen in VCN (WT = -0.022 ± 0.023, n = 8; KO = 0.009 ± 0.012, n = 10, *p* = 0.2575) or LSO (WT = -0.024 ± 0.036, n = 7, KO = -0.047 ± 0.033, *p* = 0.6444). In contrast, MNTB in *Fmr1* ^-/-^ mice revealed a reduced I_SP_ relative to control (WT = 0.51 ± 0.031, n = 9; KO = 0.19 ± 0.06, n = 11; *p* = 0.0003), suggesting greater VGAT expression relative to VGLUT expression than in wild type animals.

## DISCUSSION

Individuals with FXS demonstrate a variety of difficulties in auditory processing [11–13, 15, 16, 18, 34]. The ABR is a non-invasive test that reliably describes auditory brainstem function. Given their ease of use, ABRs may also serve as a metric for gauging the efficacy of possible treatments or therapies for auditory processing disorders, such as those seen in FXS. *Fmr1* ^-/-^ mice replicate many symptoms of FXS, including auditory processing dysfunction within the cortex [35–37], and might therefore serve as model for FXS-related anomalies in auditory processing. In this study, we performed ABR tests on *Fmr1* ^-/-^ mice, examined cell distribution in the auditory brainstem nuclei, and quantified excitatory and inhibitory inputs in these nuclei.

In the ABR measurements we found heightened response thresholds in *Fmr1* ^-/-^ mice compared to wild type mice in response to both click and tone stimuli. Peak I amplitudes were reduced in *Fmr1* ^-/-^ mice, and gain with respect to stimulus intensity was reduced compared to wild types. These observations suggest a loss of auditory function originating in the periphery. In addition, we found that peak III amplitude was also reduced in *Fmr1* ^-/-^ mice, possibly reflecting the observed decrease in peripheral input. This effect may also signify impairment in brainstem function, as peak III is thought to reflect activity in the superior olivary complex [38–40]. In contrast to ABR studies performed on human participants with FXS, we did not find evidence for differences in peak latency between wild type and in *Fmr1* ^-/-^ mice. A lack of latency effect may be attributable to the reduced size of the mouse pathway and/or to differences between the species in their auditory processing or in their response to mutations in *Fmr1*.

Consistent with a central auditory defect, we found that *Fmr1* ^-/-^ mice showed reduced cell area in VCN and MNTB. Reduced cell size in auditory nuclei has been reported in human anatomical studies [22–24] and FMRP protein expression has been demonstrated in the human auditory brainstem nuclei [27]. In addition, we showed that VGAT expression was increased in MNTB in *Fmr1* ^-/-^ mice, suggesting that cells in this nucleus receive more inhibitory inputs than in wild type animals. An increase in VGAT relative to VGLUT expression was seen in MNTB but not in VCN or LSO. Enhancement of inhibition in MNTB is potentially related to the observed reduction in ABR amplitude for peak III. Because MNTB is a sign-reversing relay nucleus, an interesting possibility is that MNTB cells receive less synaptic drive and thus provide less inhibition at subsequent stages of processing.

Individuals with FXS demonstrate generally exaggerated responses to sensory stimuli [9] and unusual cortical activity in response to sound [11–13, 15, 34] In addition to increased responses to sensory stimuli, findings from pre-pulse inhibition testing show both individuals with FXS and *Fmr1* ^-/-^ mice experience deficits in sensorimotor gating [41, 42]. Children with FXS experience corticotemporal seizures [43]. These observations, together with reports that *Fmr1* ^-/-^ mice experience audiogenic seizures and produce a greater cortical response to sound [35], suggests greater excitability in the auditory pathways.

While increased excitability might arise from early auditory regions, cochlear hearing loss has been shown to elicit central increases in gain, which may lead to hyperacusis [44, 45]. In *Fmr1* ^-/-^ mice, hyperacusis might thus originate at the level of the superior olivary complex or at later processing areas, and might develop as a consequence of gain increases that compensate for the reduced input. Similarly, audiogenic seizures likely originate at higher processing levels. Previous studies of audiogenic seizures in *Fmr1* ^-/-^ mice have demonstrated the involvement of dorsal nucleus of the lateral lemniscus and posterior intralaminar nucleus of the thalamus [30]. The lateral lemniscus and inferior colliculus comprise the fourth and fifth ABR peaks, respectively [38, 40], which no longer showed amplitude decreases in *Fmr1* ^-/-^ mice in the present study.

Increased excitability is thought to be a major factor in the symptoms presented in FXS [46–49], and modulation of the GABAergic system remains a target for therapeutic treatment [50]. Nevertheless, excessive excitation is not ubiquitous in all brain regions. Notably, several studies report enhanced long term depression in the hippocampus of *Fmr1* ^-/-^ mice [3, 51–53]. Overactive mGluR1/5 coupled with a loss of FMRP regulation initiates a biochemical cascade that results in accelerated removal of AMPA receptors from the postsynaptic membrane.

Evidence of alterations in GABAergic input in *Fmr1* ^-/-^ mice have also been reported in the striatum, cortex, brainstem, and diencephalon [54]. Normal glutamatergic synaptic transmission was found within the striatum of *Fmr1* ^-/-^ mice, but neurotransmitter release was elevated at GABAergic terminals, resulting in an increase in sIPSCs and mIPSCs. Increased expression of GAD65 and GAD67 was found in the cortex, hippocampus, brainstem, and diencephalon, suggesting excessive GABA production in those brain regions. However, areas demonstrating increased GAD65 and GAD67 also show a decrease in the GABA_A_R subunit β, which is required to form functional GABA_A_Rs.

The MNTB is a major source of inhibitory input to the superior olivary complex. The ventral cochlear nucleus sends glutamatergic input to the MNTB through the calyces of Held, and the MNTB in turn delivers glycinergic input to the LSO [55, 56]. The MNTB supplies essential inhibitory input for processing differences in sound intensity and timing. Notably, MNTB function is known to be impaired in *Fmr1* ^-/-^ mice. Kv3.1 voltage-dependent potassium channels allow extremely rapid repolarization of neurons following action potentials, and therefore enable neurons to produce many action potentials very quickly. Kv3.1 is typically distributed in a gradient within MNTB that follows the tonotopic gradient. In *Fmr1* ^-/-^ mice, Kv3.1 is not expressed along a gradient; rather expression is uniform throughout MNTB. The loss of Kv3.1 expression gradient results in an inability to follow high frequency stimulus [25]. The sodium-activated potassium channel Slack is also highly expressed in MNTB [57], where it improves temporal fidelity. FMRP binds the cytoplasmic C-terminal of Slack, and can stimulate Slack channel activity. Loss of FMRP results in greatly reduced potassium current and may thus impair the temporal precision of action potentials [57]. Coupled with our discovery of increased VGAT expression within MNTB, these findings may explain a decrease in synchrony of population activity, which may manifest as an elevation in ABR threshold.

In addition to imbalances in inhibitory and excitatory input, individuals with FXS also display changes in white and gray matter that may affect the degree and timing of population responses within different nuclei. FXS patients consistently exhibit increases in caudate nucleus, thalamus, and parietal lobe volume [58–61]. Temporal lobe white matter and frontostriatal tracts are also more extensive in FXS [61, 62]. Of particular interest, both total brainstem volume and brainstem white matter volume are enhanced in FXS individuals [59]. FXS is associated with widespread effects on cell size, cell number, fiber tract volume, and fiber density. In the auditory brainstem, alterations in cell and dendritic morphology (Kulesza et al., 2010; Beebe et al., 2014) suggest substrates for the effects of FMRP reduction on auditory function in individuals with FXS.

Our results show elevated ABR thresholds, decreased peak I and III amplitudes, and enhanced inhibition to MNTB in a mouse model of FXS. These observations provide evidence that auditory brainstem function is altered by inactivation of *Fmr1*. While central deficits may arise as a consequence of peripheral deficits, FXS may produce several independent effects. Given the diversity of FMRP targets, including those known to occur in the brainstem, our observation of both peripheral and central auditory effects in *Fmr1* ^-/-^ mice suggests that the mutation acts at numerous points along the auditory pathway.

## Supporting Information

S1 Table P values for effects on ABR input-output functions. (DOCX)

## References

[1] Ascano M, Mukherjee N, Bandaru P, Miller JB, Nusbaum JD, et al (2012) FMRP targets distinct mRNA sequence elements to regulate protein expression. Nature 492: 382–386. 10.1038/nature11737 23235829 PMC3528815

[2] Suhl JA, Chopra P, Anderson BR, Bassell GJ, Warren ST (2014) Analysis of FMRP mRNA target datasets reveals highly associated mRNAs mediated by G-quadruplex structures formed via clustered WGGA sequences. Hum Mol Genet 23: 5479–5491. 10.1093/hmg/ddu272 24876161 PMC4168832

[3] Bassell GJ, Warren ST (2008) Fragile X syndrome: loss of local mRNA regulation alters synaptic development and function. Neuron 60: 201–214. 10.1016/j.neuron.2008.10.004 18957214 PMC3691995

[4] Berry-Kravis E (2002) Epilepsy in fragile X syndrome. Developmental Medicine and Child Neurology 44: 724–728. 10.1017/S0012162201002833 12418611

[5] Farzin F, Perry H, Hessl D, Loesch D, Cohen J, et al (2006) Autism spectrum disorders and attention-deficit/hyperactivity disorder in boys with the fragile X premutation. Journal of Developmental and Behavioral Pediatrics 27: S137–S144. 10.1097/00004703-200604002-00012 16685180

[6] Hagerman R, Goodlin-Jones BL, Spence S, Albrect L, Bacalman S, et al (2002) The fragile X premutation and autistic spectrum disorders. American Journal of Human Genetics 71: 287–287.

[7] Hinton VJ, Brown WT, Wisniewski K, Rudelli RD (1991) Analysis of neocortex in 3 males with the fragile-x syndrome. American Journal of Medical Genetics 41: 289–294. 10.1002/ajmg.1320410306 1724112

[8] Irwin SA, Swain RA, Christmon CA, Chakravarti A, Weiler IJ, et al (2000) Evidence for altered Fragile-X mental retardation protein expression in response to behavioral stimulation. Neurobiology of Learning and Memory 73: 87–93. 10.1006/nlme.1999.3914 10686126

[9] Miller LJ, McIntosh DN, McGrath J, Shyu V, Lampe M, et al (1999) Electrodermal responses to sensory stimuli in individuals with fragile X syndrome: A preliminary report. American Journal of Medical Genetics 83: 268–279. 10.1002/(SICI)1096-8628(19990402)83:4<268::AID-AJMG7>3.3.CO;2-B 10208160

[10] Hagerman PJ, Greco CM, Hagerman RJ, Tassone F, Chudley AE, et al (2002) Neuronal intranuclear inclusions in a tremor/ataxia syndrome among fragile X premutation carriers. American Journal of Human Genetics 71: 259–259.

[11] Rojas DC, Benkers TL, Rogers SJ, Teale PD, Reite ML, et al (2001) Auditory evoked magnetic fields in adults with fragile X syndrome. Neuroreport 12: 2573–2576. 10.1097/00001756-200108080-00056 11496151

[12] Castren M, Paakkonen A, Tarkka IM, Ryynanen M, Partanen J (2003) Augmentation of auditory N1 in children with fragile X syndrome. Brain Topography 15: 165–171. 10.1023/A:1022606200636 12705812

[13] St Clair DM, Blackwood DH, Oliver CJ, Dickens P (1987) P3 abnormality in fragile X syndrome. Biol Psychiatry 22: 303–312. 10.1016/0006-3223(87)90148-X 2949781

[14] Van der Molen MJ, Van der Molen MW, Ridderinkhof KR, Hamel BC, Curfs LM, et al (2012) Attentional set-shifting in fragile X syndrome. Brain Cogn 78: 206–217. 10.1016/j.bandc.2011.12.008 22261226

[15] Van der Molen MJ, Van der Molen MW, Ridderinkhof KR, Hamel BC, Curfs LM, et al (2012) Auditory and visual cortical activity during selective attention in fragile X syndrome: a cascade of processing deficiencies. Clin Neurophysiol 123: 720–729. 10.1016/j.clinph.2011.08.023 21958658

[16] Arinami T, Sato M, Kondo I (1988) Auditory brain-stem responses in the fragile-x syndrome. Japanese Journal of Human Genetics 33: 224–224. PMC1715284 3376943

[17] Gillberg C, Persson E, Wahlstrom J (1986) The autism-fragile-x syndrome (afrax)—a population-based study of 10 boys. Journal of Mental Deficiency Research 30: 27–39. 3701848 10.1111/j.1365-2788.1986.tb01295.x

[18] Ferri R, Bergonzi P, Colognola RM, Musumeci SA, Petrella MA, et al (1987) Brain-stem auditory and visual evoked-potentials in fragile-x mental-retardation syndrome subjects. International Journal of Neuroscience 34: 172–172.

[19] Wisniewski KE, Segan SM, Miezejeski CM, Sersen EA, Rudelli RD (1991) THE FRA(X) SYNDROME—NEUROLOGICAL, ELECTROPHYSIOLOGICAL, AND NEUROPATHOLOGICAL ABNORMALITIES. American Journal of Medical Genetics 38: 476–480. 10.1002/ajmg.1320380267 2018089

[20] Roberts J, Hennon EA, Anderson K, Roush J, Gravel J, et al (2005) Auditory brainstem responses in young males with fragile X syndrome. Journal of Speech Language and Hearing Research 48: 494–500. 10.1044/1092-4388(2005/034) 15989407

[21] Miezejeski CM, Heaney G, Belser R, Brown WT, Jenkins EC, et al (1997) Longer brainstem auditory evoked response latencies of individuals with fragile X syndrome related to sedation. American Journal of Medical Genetics 74: 167–171. 10.1002/(SICI)1096-8628(19970418)74:2<167::AID-AJMG10>3.0.CO;2-G 9129717

[22] Kulesza RJ, Mangunay K (2008) Morphological features of the medial superior olive in autism. Brain Res 1200: 132–137. 10.1016/j.brainres.2008.01.009 18291353

[23] Lukose R, Schmidt E, Wolski TP, Murawski NJ, Kulesza RJ (2011) Malformation of the superior olivary complex in an animal model of autism. Brain Res 1398: 102–112. 10.1016/j.brainres.2011.05.013 21636076

[24] Kulesza RJ, Lukose R, Stevens LV (2011) Malformation of the human superior olive in autistic spectrum disorders. Brain Res 1367: 360–371. 10.1016/j.brainres.2010.10.015 20946889

[25] Strumbos JG, Brown MR, Kronengold J, Polley DB, Kaczmarek LK (2010) Fragile X Mental Retardation Protein Is Required for Rapid Experience-Dependent Regulation of the Potassium Channel Kv3.1b. Journal of Neuroscience 30: 10263–10271. 10.1523/JNEUROSCI.1125-10.2010 20685971 PMC3485078

[26] Wang Y, Sakano H, Beebe K, Brown MR, de Laat R, et al (2014) Intense and specialized dendritic localization of the fragile X mental retardation protein in binaural brainstem neurons: A comparative study in the alligator, chicken, gerbil, and human. J Comp Neurol 522: 2107–2128. 10.1002/cne.23520 24318628 PMC5564206

[27] Beebe K, Wang Y, Kulesza R (2014) Distribution of fragile X mental retardation protein in the human auditory brainstem. Neuroscience 273: 79–91. 10.1016/j.neuroscience.2014.05.006 24838064

[28] Galvez R, Gopal AR, Greenough WT (2003) Somatosensory cortical barrel dendritic abnormalities in a mouse model of the fragile X mental retardation syndrome. Brain Research 971: 83–89. 10.1016/S0006-8993(03)02363-1 12691840

[29] Pan LY, Zhang YQ, Woodruff E, Broadie K (2004) The Drosophila fragile X gene negatively regulates neuronal elaboration and synaptic differentiation. Current Biology 14: 1863–1870. 10.1016/j.cub.2004.09.085 15498496

[30] Chen L, Toth M (2001) Fragile X mice develop sensory hyperreactivity to auditory stimuli. Neuroscience 103: 1043–1050. 10.1016/S0306-4522(01)00036-7 11301211

[31] Irwin SA, Idupulapati M, Gilbert ME, Harris JB, Chakravarti AB, et al (2002) Dendritic spine and dendritic field characteristics of layer V pyramidal neurons in the visual cortex of fragile-X knockout mice. American Journal of Medical Genetics 111: 140–146. 10.1002/ajmg.10500 12210340

[32] Rotschafer SE, Trujillo MS, Dansie LE, Ethell IM, Razak KA (2012) Minocycline treatment reverses ultrasonic vocalization production deficit in a mouse model of Fragile X Syndrome. Brain Res 1439: 7–14. 10.1016/j.brainres.2011.12.041 22265702

[33] Spencer CM, Alekseyenko O, Serysheva E, Yuva-Paylor LA, Paylor R (2005) Altered anxiety-related and social behaviors in the Fmr1 knockout mouse model of fragile X syndrome. Genes Brain Behav 4: 420–430. 10.1111/j.1601-183X.2005.00123.x 16176388

[34] Van der Molen MJ, Van der Molen MW, Ridderinkhof KR, Hamel BC, Curfs LM, et al (2012) Auditory change detection in fragile X syndrome males: a brain potential study. Clin Neurophysiol 123: 1309–1318. 10.1016/j.clinph.2011.11.039 22192499

[35] Rotschafer S, Razak K (2013) Altered auditory processing in a mouse model of fragile X syndrome. Brain Res 1506: 12–24. 10.1016/j.brainres.2013.02.038 23458504

[36] Rotschafer SE, Razak KA (2014) Auditory processing in fragile x syndrome. Front Cell Neurosci 8: 19 10.3389/fncel.2014.00019 24550778 PMC3912505

[37] Kim H, Gibboni R, Kirkhart C, Bao S (2013) Impaired critical period plasticity in primary auditory cortex of fragile X model mice. J Neurosci 33: 15686–15692. 10.1523/JNEUROSCI.3246-12.2013 24089476 PMC3787494

[38] Melcher JR (1996) Cellular generators of the binaural difference potential in cat. Hear Res 95: 144–160. 10.1016/0378-5955(96)00032-9 8793516

[39] K P, XM S, DO K (2001) Noninvasive assessment of auditory function in mice: auditory brainstem response and distortion product otoacoustic emissions. In: Willott JF, editor. Handbook of Mouse Auditory Research: From Behavior to Molecular Biology. Boca Raton, FL: CRC Press pp. 37–58.

[40] Willott JF (2001) Handbook of mouse auditory research: from behavior to molecular biology.

[41] Nielsen DM, Derber WJ, McClellan DA, Crnic LS (2002) Alterations in the auditory startle response in Fmr1 targeted mutant mouse models of fragile X syndrome. Brain Res 927: 8–17. 10.1016/S0006-8993(01)03309-1 11814427

[42] Frankland PW, Wang Y, Rosner B, Shimizu T, Balleine BW, et al (2004) Sensorimotor gating abnormalities in young males with fragile X syndrome and Fmr1-knockout mice. Mol Psychiatry 9: 417–425. 10.1038/sj.mp.4001432 14981523

[43] Heard TT, Ramgopal S, Picker J, Lincoln SA, Rotenberg A, et al. (2014) EEG abnormalities and seizures in genetically diagnosed Fragile X syndrome. Int J Dev Neurosci. 10.1016/j.ijdevneu.2014.07.002 25016068

[44] Knipper M, Van Dijk P, Nunes I, Rüttiger L, Zimmermann U (2013) Advances in the neurobiology of hearing disorders: recent developments regarding the basis of tinnitus and hyperacusis. Prog Neurobiol 111: 17–33. 10.1016/j.pneurobio.2013.08.002 24012803

[45] Zeng FG (2013) An active loudness model suggesting tinnitus as increased central noise and hyperacusis as increased nonlinear gain. Hear Res 295: 172–179. 10.1016/j.heares.2012.05.009 22641191 PMC3593089

[46] Olmos-Serrano JL, Paluszkiewicz SM, Martin BS, Kaufmann WE, Corbin JG, et al (2010) Defective GABAergic neurotransmission and pharmacological rescue of neuronal hyperexcitability in the amygdala in a mouse model of fragile X syndrome. J Neurosci 30: 9929–9938. 10.1523/JNEUROSCI.1714-10.2010 20660275 PMC2948869

[47] Paluszkiewicz SM, Olmos-Serrano JL, Corbin JG, Huntsman MM (2011) Impaired inhibitory control of cortical synchronization in fragile X syndrome. J Neurophysiol 106: 2264–2272. 10.1152/jn.00421.2011 21795626 PMC3214096

[48] Gibson JR, Bartley AF, Hays SA, Huber KM (2008) Imbalance of neocortical excitation and inhibition and altered UP states reflect network hyperexcitability in the mouse model of fragile X syndrome. J Neurophysiol 100: 2615–2626. 10.1152/jn.90752.2008 18784272 PMC2585391

[49] Rubenstein JL, Merzenich MM (2003) Model of autism: increased ratio of excitation/inhibition in key neural systems. Genes Brain Behav 2: 255–267. 10.1034/j.1601-183X.2003.00037.x 14606691 PMC6748642

[50] Lozano R, Hare EB, Hagerman RJ (2014) Modulation of the GABAergic pathway for the treatment of fragile X syndrome. Neuropsychiatr Dis Treat 10: 1769–1779. 10.2147/NDT.S42919 25258535 PMC4172237

[51] Bear MF, Huber KM, Warren ST (2004) The mGluR theory of fragile X mental retardation. Trends Neurosci 27: 370–377. 10.1016/j.tins.2004.04.009 15219735

[52] Osterweil EK, Krueger DD, Reinhold K, Bear MF (2010) Hypersensitivity to mGluR5 and ERK1/2 leads to excessive protein synthesis in the hippocampus of a mouse model of fragile X syndrome. J Neurosci 30: 15616–15627. 10.1523/JNEUROSCI.3888-10.2010 21084617 PMC3400430

[53] Huber K (2007) Fragile X syndrome: molecular mechanisms of cognitive dysfunction. Am J Psychiatry 164: 556 10.1176/appi.ajp.164.4.556 17403966

[54] El Idrissi A, Ding XH, Scalia J, Trenkner E, Brown WT, et al (2005) Decreased GABA(A) receptor expression in the seizure-prone fragile X mouse. Neurosci Lett 377: 141–146. 10.1016/j.neulet.2004.11.087 15755515

[55] Brown MR, Kaczmarek LK (2011) Potassium channel modulation and auditory processing. Hear Res 279: 32–42. 10.1016/j.heares.2011.03.004 21414395 PMC3137660

[56] Kandler K, Gillespie DC (2005) Developmental refinement of inhibitory sound-localization circuits. Trends Neurosci 28: 290–296. 10.1016/j.tins.2005.04.007 15927684 PMC4120098

[57] Brown MR, Kronengold J, Gazula VR, Chen Y, Strumbos JG, et al (2010) Fragile X mental retardation protein controls gating of the sodium-activated potassium channel Slack. Nature Neuroscience 13: 819–821. 10.1038/nn.2563 20512134 PMC2893252

[58] Hoeft F, Walter E, Lightbody AA, Hazlett HC, Chang C, et al (2011) Neuroanatomical differences in toddler boys with fragile x syndrome and idiopathic autism. Arch Gen Psychiatry 68: 295–305. 10.1001/archgenpsychiatry.2010.153 21041609 PMC4369209

[59] Hallahan BP, Craig MC, Toal F, Daly EM, Moore CJ, et al (2011) In vivo brain anatomy of adult males with Fragile X syndrome: an MRI study. Neuroimage 54: 16–24. 10.1016/j.neuroimage.2010.08.015 20708694

[60] Gothelf D, Furfaro JA, Hoeft F, Eckert MA, Hall SS, et al (2008) Neuroanatomy of fragile X syndrome is associated with aberrant behavior and the fragile X mental retardation protein (FMRP). Ann Neurol 63: 40–51. 10.1002/ana.21243 17932962 PMC2773141

[61] Hazlett HC, Poe MD, Lightbody AA, Styner M, MacFall JR, et al (2012) Trajectories of early brain volume development in fragile X syndrome and autism. J Am Acad Child Adolesc Psychiatry 51: 921–933. 10.1016/j.jaac.2012.07.003 22917205 PMC3428739

[62] Haas BW, Barnea-Goraly N, Lightbody AA, Patnaik SS, Hoeft F, et al (2009) Early white-matter abnormalities of the ventral frontostriatal pathway in fragile X syndrome. Dev Med Child Neurol 51: 593–599. 10.1111/j.1469-8749.2009.03295.x 19416325 PMC2715437

