# Supplementary material for: Deletion of Fmr1 Alters Function and Synaptic Inputs in the Auditory Brainstem
Source: PLoS One. 2015 Feb 13;10(2):e0117266. doi: 10.1371/journal.pone.0117266 (PMC4332492; doi:10.1371/journal.pone.0117266)
Supplement: S1 Table — (DOCX) [file pone.0117266.s001.docx]

| **Table S1. *P* values for effects on ABR input-output functions** | | | | | | | |
| --- | --- | --- | --- | --- | --- | --- | --- |
|  | **Peak Amplitude** | |  | | **Peak Latency** | | |
| ***Stimulus*** | **Genotype** | **dB** | | **Genotype**  **x dB** | **Genotype** | **dB** | **Genotype**  **x dB** |
| *Click peak I* | 0.0193* | 0.0001* | | 0.0087* | 0.6422 | 0.001* | 0.2929 |
| *8kHz peak I* | 0.0131* | 0.0001* | | 0.2053 | 0.2897 | 0.001* | 0.4154 |
| *12kHz peak I* | 0.0019* | 0.0001* | | 0.0433* | 0.4130 | 0.0001* | 0.1909 |
| *16kHz peak I* | 0.0029* | 0.0001* | | 0.1469 | 0.1276 | 0.0001* | 0.4232 |
| *24kHz peak I* | 0.7180 | 0.1482 | | 0.9077 | 0.4255 | 0.0064* | 0.0148* |
| *32kHz peak I* | 0.9086 | 0.0153* | | 0.1309 | 0.3394 | 0.0409* | 0.6451 |
|  |  |  | |  |  |  |  |
| *Click peak II* | 0.0511 | 0.0001* | | 0.4975 | 0.5056 | 0.0001* | 0.3290 |
| *8kHz peak II* | 0.0721 | 0.0001* | | 0.3946 | 0.6007 | 0.0001* | 0.1333 |
| *12kHz peak II* | 0.0516 | 0.0001* | | 0.0251* | 0.6797 | 0.0001* | 0.9324 |
| *16kHz peak II* | 0.0628 | 0.0001* | | 0.1083 | 0.0144* | 0.0106* | 0.3762 |
| *24kHz peak II* | 0.1764 | 0.0360* | | 0.2468 | 0.1764 | 0.0360* | 0.2468 |
| *32kHz peak II* | 0.0530 | 0.0005* | | 0.0377* | 0.3008 | 0.0343* | 0.0383* |
|  |  |  | |  |  |  |  |
| *Click peak III* | 0.0039* | 0.0001* | | 0.1060 | 0.6699 | 0.0086* | 0.1968 |
| *8kHz peak III* | 0.0112* | 0.0004* | | 0.1044 | 0.4649 | 0.0224* | 0.4040 |
| *12kHz peak III* | 0.1074 | 0.0103* | | 0.1830 | 0.0092* | 0.0009* | 0.0050* |
| *16kHz peak III* | 0.5354 | 0.2104 | | 0.7650 | 0.8522 | 0.2241 | 0.5727 |
| *24kHz peak III* | 0.6362 | 0.0011* | | 0.3508 | 0.7755 | 0.4947 | 0.3463 |
|  |  |  | |  |  |  |  |
| *Click peak IV* | 0.1018 | 0.0823 | | 0.7807 | 0.5008 | 0.9858 | 0.9688 |
| *8kHz peak IV* | 0.5934 | 0.3753 | | 0.8156 | 0.7405 | 0.0567 | 0.0497* |
| *12kHz peak IV* | 0.4274 | 0.1607 | | 0.5647 | 0.5639 | 0.0953 | 0.6332 |
| *16kHz peak IV* | 0.0314* | 0.2656 | | 0.4824 | 0.5404 | 0.3397 | 0.5065 |
|  |  |  | |  |  |  |  |
| *Click peak V* | 0.3510 | 0.6464 | | 0.9118 | 0.5745 | 0.0989 | 0.4144 |
|  |  |  | |  |  |  |  |
